# Efficacy and Safety of High-Dose Chemotherapy with Treosulfan and Melphalan in Multiple Myeloma

**DOI:** 10.3390/cancers15102699

**Published:** 2023-05-10

**Authors:** Cédric Gillich, Dilara Akhoundova, Michael Hayoz, Yolanda Aebi, Carlo R. Largiadèr, Katja Seipel, Michael Daskalakis, Ulrike Bacher, Thomas Pabst

**Affiliations:** 1 Department of Medical Oncology, University Hospital Inselspital, University of Bern, 3010 Bern, Switzerland; 2 Department of Clinical Chemistry and Center for Laboratory Medicine, University Hospital Inselspital, University of Bern, 3010 Bern, Switzerland; 3 University Institute of Clinical Chemistry, University Hospital Inselspital, University of Bern, 3010 Bern, Switzerland; 4 Department of Hematology, University Hospital Inselspital, University of Bern, 3010 Bern, Switzerland

**Keywords:** multiple myeloma (MM), treosulfan, pharmacokinetics, efficacy, response rate, overall survival (OS), progression-free survival (PFS), high risk, standard risk

## Abstract

**Simple Summary:**

Treatment consolidation using high-dose chemotherapy (HDCT) and autologous stem cell transplantation (ASCT) has relevantly contributed to achieving durable remissions in multiple myeloma (MM) patients. The optimization of HDCT regimens can, therefore, essentially contribute to further improving the depth and duration of tumor remissions. In our previous work, we showed that the combination of treosulfan and melphalan (TreoMel) was effective and safe as a conditioning regimen in acute myeloid leukemia (AML) patients undergoing ASCT. Based on these data, TreoMel has been adopted as the standard of care for fit MM patients at our institution. In the current work, we analyzed data from 115 MM patients who underwent consolidation via TreoMel and ASCT between 01/2020 and 08/2022 at the University Hospital of Bern. We report a promising complete response (CR) rate of 84%, which is comparable to the CR rate achieved for the quadruplet combination. The median progression-free survival (PFS) was 30 months (95% CI: 20.4—not reached) and the 31-month overall survival (OS) rate was 83%. The results from our study suggest that TreoMel should be further explored as a conditioning regimen for first-line HDCT consolidation in MM.

**Abstract:**

(1) Background: Upfront treatment consolidation with high-dose chemotherapy (HDCT) and autologous stem cell transplantation (ASCT) has relevantly contributed to achieving durable remissions following induction treatment in multiple myeloma (MM) patients. The optimization of HDCT regimens can, therefore, essentially contribute to improving the depth and duration of tumor remissions. To date, melphalan at 200 mg/m^2^ is the standard HDCT regimen for fit MM patients. In our previous work, we showed promising efficacy and safety results for treosulfan (14 g/m^2^) and melphalan (200 mg/m^2^) (TreoMel) in acute myeloid leukemia (AML) patients receiving ASCT. Based on these data, TreoMel became the standard of care for fit MM patients at our institution. (2) Methods: We identified 115 consecutive MM patients who underwent consolidation with TreoMel between 01/2020 and 08/2022 at the University Hospital of Bern. We analyzed the safety and efficacy data, as well as the treosulfan pharmacokinetics, correlating them with tumor responses. (3) Results: A complete response (CR) rate of 84% was achieved, which is comparable to the CR rate reported for the quadruplet combination. The median PFS was 30 months (95% CI: 20.4—not reached), and the 31-month OS rate was 83%. The median area under the curve (AUC) for treosulfan was 952.5 mg*h/L (range: 527.4–1781.4), and the median peak level was 332.3 mg/L (range: 168–554). The treosulfan pharmacokinetics showed no significant correlation with MM responses after HDCT and ASCT. However, female patients had a significantly higher AUC (*p* = 0.007) and peak value (*p* = 0.001), and the higher values were associated with longer hospitalizations. (4) Conclusions: Treatment consolidation with TreoMel HDCT demonstrated a promising efficacy and safety profile in our cohort of MM patients and deserves further investigation in prospective studies.

## 1. Introduction

Multiple myeloma (MM) is a plasma cell-derived malignancy clinically characterized by a prolonged disease course and recurrent relapses. The typical disease features are osteolytic lesions, anemia, renal impairment, hypercalcemia, and an increased risk of infections. MM is slightly more common in male patients (58%), and the median age at presentation is around 60 years. Along with increasing life expectancy, the incidence of MM has been continuously rising over the last two decades. Immunomodulatory drugs, proteasome inhibitors, and anti-CD38 antibodies constitute the backbone of the systemic treatment of MM. Since the late 1990s, high-dose chemotherapy (HDCT) with melphalan followed by autologous stem cell transplantation (ASCT) has become the standard for fit patients [1,2,3,4,5,6,7,8,9]. A recent analysis of a cohort of 61 elderly MM patients suggested that HDCT with ASCT might also be safe and effective in elderly patients who are fit enough [10]. The longer-term follow-up of the IFM 2009 study showed that HDCT with melphalan at 200 mg/m^2^ and ASCT significantly improved progression-free survival (PFS) after first-line induction treatment in patients with MM, albeit without impact on overall survival (OS). This finding was also confirmed by the more recent data from the DETERMINATION trial, where upfront treatment consolidation with HDCT and ASCT led to improved PFS but not OS in a similar patient population. The IFM 2009 randomized study also showed that HDCT with melphalan and bortezomib was not superior to melphalan at 200 mg/m^2^ alone [10,11,12,13].

Globally, the median OS of MM patients has relevantly improved over recent years due to broader access to more effective treatment combinations, including CD38-targeting antibodies. However, disease relapse after first-line induction and consolidation treatment still occurs in the majority of patients, and disease progression, despite subsequent treatment regimens, remains the major cause of death [14,15,16,17,18]. Therefore, improving the duration and depth of tumor responses following first-line induction and HDCT consolidation remains a relevant clinical need. Currently, melphalan at 200 mg/m^2^ constitutes the standard of care for HDCT in MM patients. We previously reported that the addition of bendamustine (BenMel) increases the rate of stringent complete responses (sCR) and complete responses (CR) from 51.7% to 70% but also leads to an increased incidence of reversible renal toxicity [15,18].

To the best of our knowledge, no data regarding the efficacy and safety of treosulfan-based HDCT previous to ASCT are available for MM patients. In a retrospective study, treosulfan single-drug conditioning before first-line allogenic stem cell transplantation showed improved OS when compared to myeloablative conditioning regimens [19]. In previous work, our group assessed the efficacy and safety of TreoMel in a cohort of acute myeloid leukemia (AML) patients undergoing ASCT, showing comparable efficacy and safety results to HDCT with BuMel [20]. Moreover, no cases of irreversible alopecia and less incidence of neurologic toxicity were observed with the TreoMel regimen [20]. Treosulfan is a water-soluble, bifunctional, and alkylating prodrug that undergoes a pH- and temperature-dependent non-enzymatic conversion into an active drug [20]. The active drug is then predominantly eliminated by glomerular filtration and has a tubular reabsorption rate of ~60% [20,21,22]. Current evidence suggests that treosulfan is associated with lower rates of early liver, lung, and neurological toxicity when compared to busulfan-based conditioning [23]. Treosulfan has been approved by the European Medicines Agency as a conditioning agent prior to stem cell transplantation.

Based on our own previous AML data [20], TreoMel has been adopted as the standard of care HDCT regimen for fit MM patients at our institution. In this study, we retrospectively assessed the performance of treosulfan at 14 g/m^2^ (day −4 to −2) and melphalan at 200 mg/m^2^ (day −1) as an HDCT regimen in a large cohort of MM patients. To our knowledge, this is the first study analyzing the performance of TreoMel HDCT in this setting.

## 2. Materials and Methods

### 2.1. Study Design, Endpoints, and Patient Cohort

We performed a retrospective analysis of a cohort of MM patients who received first-line HDCT consolidation with TreoMel followed by ASCT. The main endpoints of the study were the efficacy (response rate, PFS, and OS) and safety of HDCT with TreoMel. The secondary endpoints were the treosulfan pharmacokinetics, as well as their correlation with response and toxicity.

The patients included in the study were treated at the University Hospital of Bern, Switzerland, between January 2020 and August 2022. The study was performed following the Declaration of Helsinki and was approved by the local ethics committee of Bern, Switzerland: Ethics Commission of the Canton of Bern (decision number #2022-01923; date of approval: 20 January 2023). All patients signed written informed consent.

### 2.2. HDCT Treatment Schedule

Following induction therapy and stem cell mobilization and apheresis, all patients received treosulfan at 14 g/m^2^ on days −4, −3, and −2, followed by melphalan at 200 mg/m^2^ on day 1, before ASCT (day 0). Treosulfan was administered intravenously (iv) in a 5% glucose solution over 2 h, and melphalan in a 0.9% NaCl solution iv over 1 h. On day 0, all patients received autologous stem cell reinfusion. Patients received sulfamethoxazole-trimethoprim, fluconazol, and valaciclovir as anti-infective prophylaxis. Dexamethasone was administered on days −4 to 0 and +9 to +13 to prevent engraftment syndrome. All patients received allopurinol during HDCT and premedication with methylprednisolone and clemastine previous to stem cell reinfusion. Following ASCT, zoledronic acid was administered at day +1, folic acid daily starting from day +1 and up to 8 weeks, as well as filgrastim 5 μg/kg/day between days +6 and +12.

### 2.3. Assessment of Treosulfan Pharmacokinetics

Treosulfan circulating levels were assessed in the peripheral blood samples collected on day −3 of the HDCT schedule. A first blood sample was collected before treosulfan infusion, and 5 further samples 30, 60, 120, 240, and 360 min after completion of the infusion. Treosulfan circulating levels were assessed in the blood samples collected on day −3 of the HDCT schedule. A first blood sample was collected before treosulfan infusion, and 5 further samples 30, 60, 120, 240, and 360 min after completion of the infusion. To assess treosulfan concentrations, we employed an ultra-performance liquid chromatography tandem mass spectrometry (UPLC-MS-MS) method. We performed the mass spectrometric measurements through multiple reaction monitoring by employing a Xevo TQ-S (Waters Corp., Milford, MA, USA), as previously described [20]. Briefly, immediately following blood collection, blood samples were stabilized by adding a sodium citrate buffer and stored at −80 °C until analysis. Solutions of treosulfan and the internal standard (^2^H_4_)-treosulfan, as well as 6 calibrator-spiking solutions at 2.8, 5.6, 11.3, 22.5, 45, and 90 mg/L, were prepared, as previously described [23]. After blood sample preparation, 0.5 μL of each sample was injected into a reverse-phase CORECTS UPLC T3 column and resolved for 3.0 min. Finally, the eluent was introduced by electrospray ionization into the mass spectrometer (Xevo TQ-S, Waters Corp., Milford, MA, USA), which operated in a positive ion electrospray ionization mode [23]. Data processing was performed using TargetLynx (MassLynx software, version 4.1, Waters Corp., Milford, MA, USA) via the integration of the area under the specific multiple reactions monitoring chromatograms compared to the area of the isotope-labeled analog [23].

### 2.4. Response Assessment

Initial staging of patients was performed according to the Revised International Staging System (R-ISS). Patients were classified as having high-risk cytogenetics if at least one of the following genomic alterations was detected: t(4;14), t(14;16), or del(17p). MM responses were assessed following the International Myeloma Working Group (IMWG) response criteria. OS was defined as the time from the start of TreoMel HDCT until death as per any cause or loss of follow-up. PFS was defined as the time from the start of TreoMel therapy until disease progression or relapse, death, or loss of follow-up, whichever occurred first.

### 2.5. Statistical Analysis

The data cut-off was on 30 August 2022. Clinical data were recorded with Microsoft Excel. Statistical analysis and graphs were performed with GraphPad Prism^®^ 8.3.0. PFS and OS were calculated and graphically represented via Kaplan–Meier survival analysis using GraphPad Prism^®^ 8.3.0. The median and percentages were rounded to whole numbers.

## 3. Results

### 3.1. Patient Baseline Characteristics

The patient characteristics at the time of diagnosis are summarized in Table 1. There were 73 (63%) males and 42 (37%) females. The median age was 61 years (range: 36–73). A total of 79 patients (69%) had an IgG-type MM, 75 (65%) a kappa-type, and 19 patients (16%) a light-chain-only-type. The R-ISS stage was calculated in 107 patients (93%), with 27 patients (25%) diagnosed as stage I, 51(48%) as stage II, and 29 (27%) as stage III. At diagnosis, 46 patients (40%) presented with anemia, 21 (18%) with hypercalcemia, 23 (20%) had renal impairment, and in 88 patients (77%), osteolytic lesions were detected. The median bone marrow infiltration was 60%, ranging from 5 to 100%. Cytogenetics (array-CGH or FISH) was available for 91 patients (79%), and 25 patients (28%) were classified as high-risk cytogenetic due to the presence of at least one of the following genomic alterations: t(4;14), t(14;16), or del(17p). 1q21 amplification (1q21+) was classified as standard risk.

### 3.2. Induction and Maintenance Therapy

A total of 107 patients (93%) received HDCT and ASCT in first-line treatment, five (4%) in second-line, and three (3%) in third-line treatment. The most frequent induction regimen was the combination of bortezomib, lenalidomide, and dexamethasone (VRD), which was administered in 98 patients (85%). Daratumumab, lenalidomide, bortezomib, and dexamethasone (Dara-RVd) were employed in 11 patients (10%); bortezomib, cyclophosphamide, and dexamethasone (VCD) in 5 patients (4%); daratumumab, lenalidomide, and dexamethasone (Dara-Rd) in three patients (3%), and daratumumab, carfilzomib, and dexamethasone (Dara-Kd) in two patients (2%). A total of 107 patients (93%) received one HDCT consolidation with TreoMel, seven patients (6%) received two, and one patient (1%) received three. Following HDCT and ASCT, 97 patients (92%) received lenalidomide maintenance, two (2%) patients received daratumumab and lenalidomide, and one patient (1%) received rituximab and lenalidomide. Five patients (5%) received no maintenance therapy (Table 2).

### 3.3. Treosulfan Pharmacokinetics

**Figure 1:**
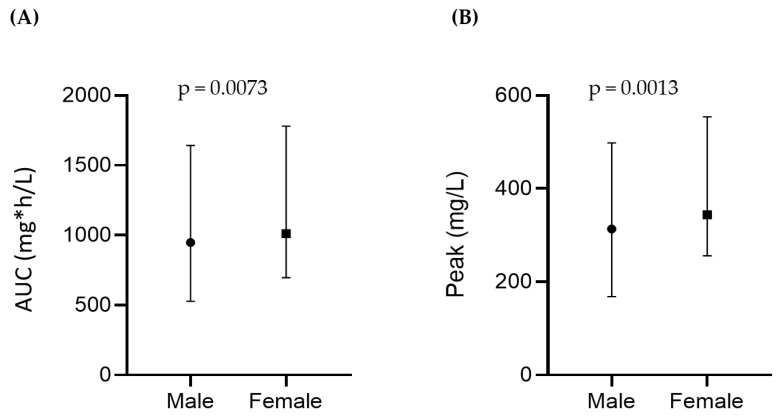

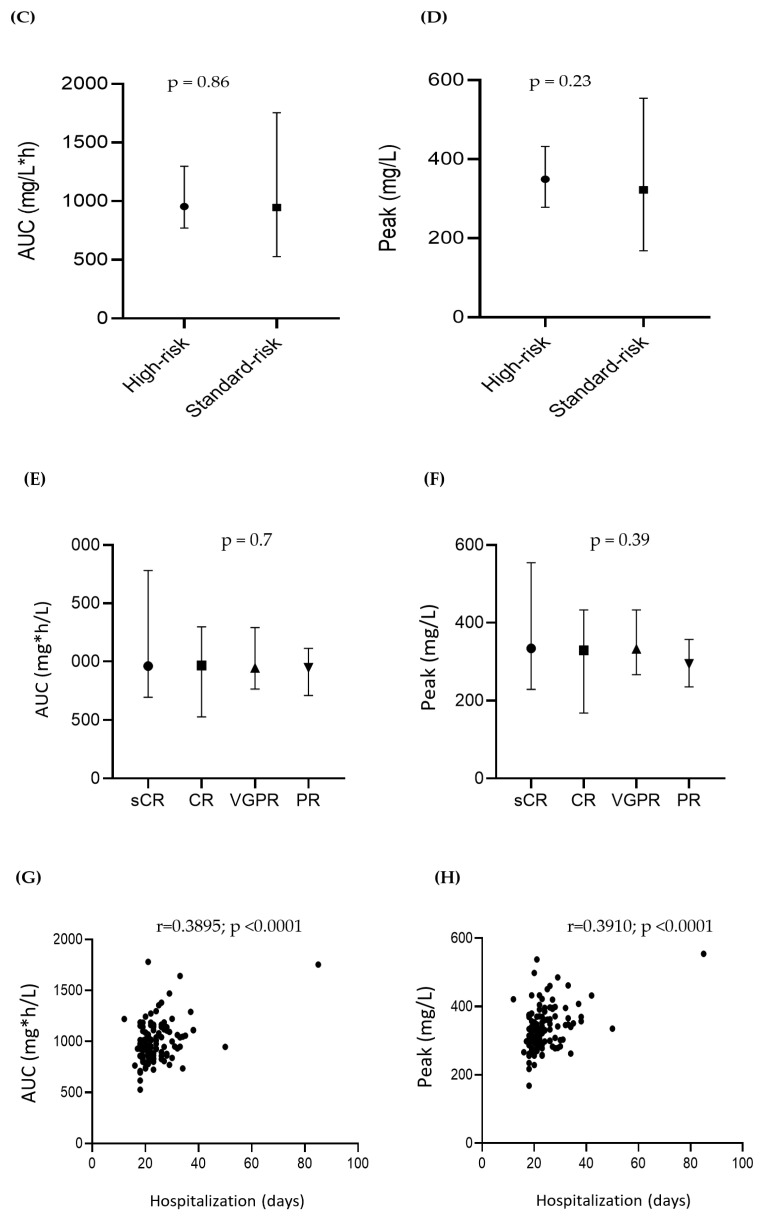
(**A**,**B**) Comparison of treosulfan AUC and peak values (median; range) in female vs. male patients; (**C**,**D**) patients with high- vs. standard-risk cytogenetics, and (**E**,**F**) remission status after HDCT and ASCT. (**G**,**H**) Correlation of treosulfan AUC and peak values with the duration of hospitalization (days). After data analysis with the exclusion of the outlier at day 85: Pearson correlation factor r = 0.23, *p* = 0.015. *: multiplicated by.

The results of the pharmacokinetic analyses are presented in Figure 1. The mean area under the curve (AUC) for the entire patient cohort was 997.5 mg*h/L (standard deviation (SD): ±202.7); the median area was 952.5 mg*h/L (range: 527.4–1781.4). The mean peak level was 336.6 mg/L (SD: ±63.9), and the median peak level was 332.3 mg/L (range: 168–554). The treosulfan AUC and peak level both positively correlated with a factor of 0.93 (*p* ≤ 0.0001, not shown). A significant difference regarding AUC and peak values was observed for female vs. male patients (Figure 1A,B). Female patients showed a median AUC of 1011.2 mg*h/L (range: 695.6–1781.4) and a median peak value of 343.8 mg/L (range: 255.5–554.3). For males, the median AUC and peak values were 948 mg*h/L (range: 527.4–1643.3) and 313 mg/L (range: 168.2–498 mg/L), respectively. No significant differences were observed for patients with high- vs. standard-risk (*n* = 66) cytogenetics (Figure 1C,D). We found no correlation between treosulfan AUC or the peak values and remission status after HDCT and ASCT (Figure 1E,F). Both AUC and the peak values showed a significant but weak positive correlation with the duration of hospitalization, with a Pearson correlation factor r = 0.3895 and r = 0.3910, respectively (*p* < 0.0001 for both parameters). This correlation remained significant after excluding the outlier at day 85 (r = 0.23, *p* = 0.015) (Figure 1G,H).

### 3.4. Adverse Events

The median duration of hospitalization was 22 days (range: 16–85), and the median duration of pancytopenia was 8 days (range: 5–24). Platelet transfusions were required in 109 patients (95%) and erythrocyte transfusions in 58 (50%). A median amount of three platelet concentrates (range: 1–30) and one erythrocyte concentrate (range: 1–19) were administered. Gastrointestinal disorders were frequent, leading to parenteral nourishment in 101 patients (88%) for a median duration of 9 days (range: 2–25). Malnutrition was observed in 100 patients (87%), neutropenic enterocolitis in 72 patients (63%), and refeeding syndrome in 44 (38%). Eight patients (7%) developed an acute kidney injury (AKI stage I in four patients, stage II in two patients, and stage III in two patients). Bacteremia occurred in 28 patients (24%), and four patients (3%) developed sepsis with a sepsis-related organ failure assessment (SOFA) score of ≥3. Four patients (3%) had to be transferred to the intensive care unit, and two patients (2%) died due to HDCT-related infectious complications. Candidosis occurred in 14 patients (12%). Chemotherapy-associated polyneuropathy was reported in 22 patients (19%). Diabetic complications, such as steroid-induced diabetes and aggravated type II diabetes, occurred in 30% of patients. Other less common complications were atrial fibrillation and hepatic disorders (Table 3).

### 3.5. Outcome of HDCT and ASCT

**Figure 2:**
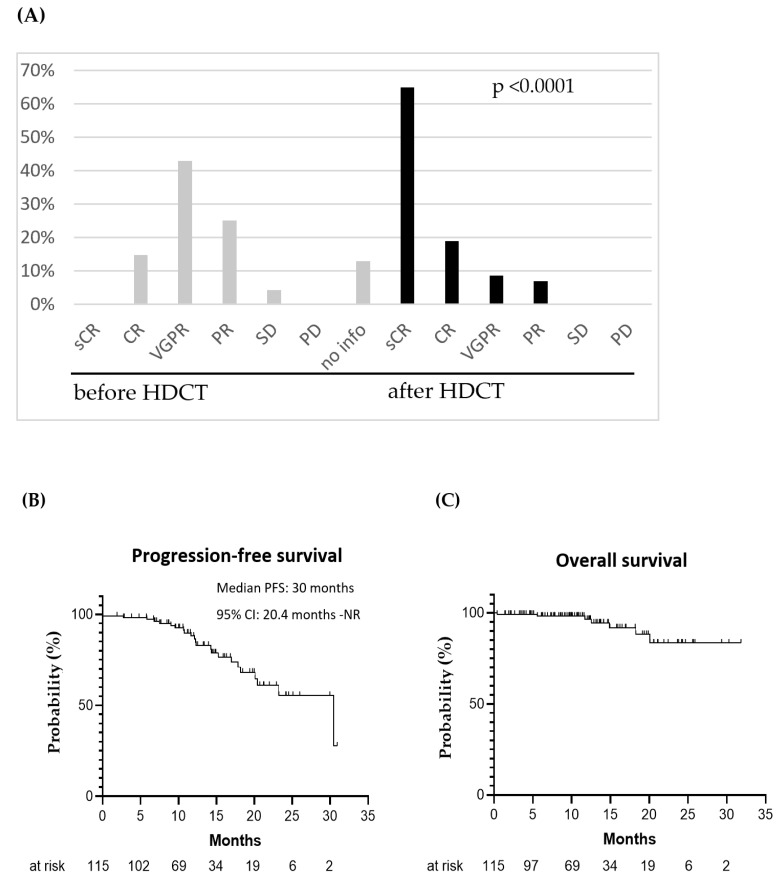
(**A**) MM remission status before and after HDCT; (**B**) Progression-free survival and (**C**) overall survival since start of HDCT (median OS could not be calculated due to data immaturity); NR: not reached.

The median follow-up since the start of TreoMel treatment was 12 months (range: 0.4–31 months). Following the induction of treatment, 16 patients (16%) had achieved CR, 50 (50%) achieved a very good partial response (VGPR), 29 (29%) achieved a partial response (PR), and 5 (5%) achieved a stable disease (SD) condition. For 15 patients, the data regarding remission status before HDCT were not available. After HDCT, 74 patients (65%) achieved sCR, 22 (19%) CR, 10 (9%) VGPR, and eight (7%) PR. No patient showed SD or progressive disease (PD) (Table 4, Figure 2A). Twenty-three patients (20%) relapsed during follow-up, and the median PFS was 30 months (95% CI: 20.4—not reached) (Figure 2B). At 31 months, the OS was 83%, and seven deaths occurred (Figure 2C). Four patients died due to disease progression, and two patients died due to HDCT-related infectious complications (mucormycosis and septic shock, respectively). One patient died due to pneumonia 14 months after HDCT, so this infection was considered to be unlikely related to HDCT.

### 3.6. Clinical Outcomes in Patients with Standard vs. High-Risk Cytogenetics

**Figure 3:**
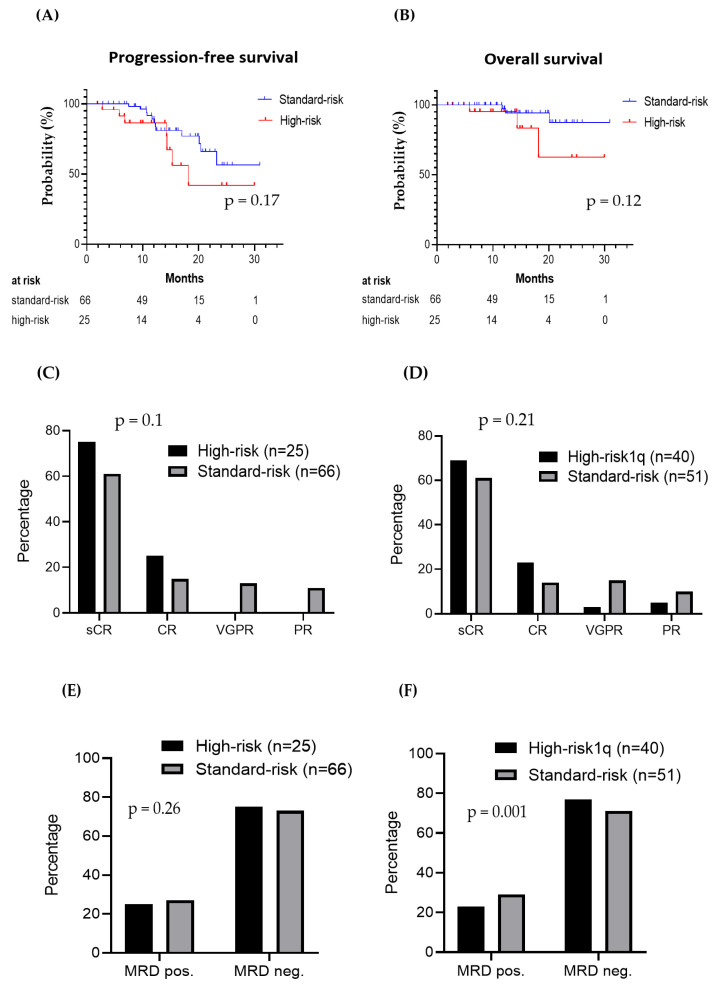

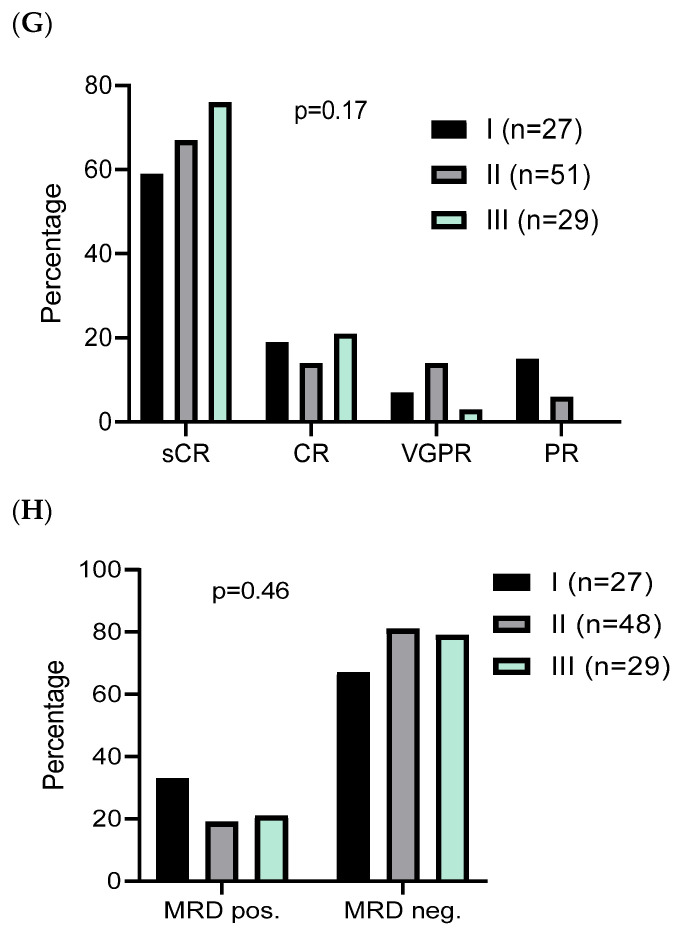
(**A**,**B**) Progression-free survival and overall survival from the start of HDCT in patients with standard- vs. high-risk cytogenetics. (**C**,**E**) Remission status and MRD status post-HDCT in patients with standard- vs. high-risk cytogenetics. (**D**,**F**) Remission status and MRD status post-HDCT in patients within standard- vs. high-risk1q cytogenetic groups (with the inclusion of patients with 1q gain). (**G**,**H**) Remission status and MRD status post-HDCT by R-ISS stage.

We compared PFS, OS, remission status, and minimal residual disease (MRD) via flow cytometry after HDCT and ASCT in patients with standard- vs. high-risk cytogenetics. The results of the cytogenetic analysis were available for 91 patients (79%); the remaining 24 patients could not be included in this analysis. Patients with del(17p), translocation t(4;14), and translocation t(14;16) were classified as high-risk. In our cohort, 66 patients had standard-risk cytogenetics and 25 high-risk cytogenetics. We observed a statistically nonsignificant trend towards better PFS (HR: 0.48, *p* = 0.17) and OS (HR: 0.21, *p* = 0.12) for those patients with standard-risk cytogenetics (Figure 3A,B). Unequal sample sizes could have potentially contributed to nonsignificant *p* values. In an additional analysis, 15 patients with ≥3 copies of 1q21 (1q21+) were reclassified within the “high-risk1q” group (*n* = 40) and were compared to the rest of the standard-risk patients (*n* = 51). We observed no significant differences in the response rates and MRD rates between both stratifications (Figure 3C–F). Similarly, the sCR rates were 60.7% vs. 66.7% vs. 75.9%, and the MRD negativity rates were 79.3% vs. 76.5% vs. 67.9% for R-ISS stage I, II, and III, respectively (Figure 3G,H).

## 4. Discussion

Melphalan at 200 mg/m^2^ is currently the standard HDCT regimen for younger MM patients [24,25]. For patients over the age of 65 or with renal impairment, a dose reduction in melphalan to 140 mg/m^2^ is recommended [24]. A randomized phase III study compared the efficacy and safety of melphalan 200 mg/m^2^ to BuMel. Conditioning with BuMel led to improvement in PFS (64.7 vs. 43.5 months, *p* = 0.022) but was associated with a significantly higher mucositis rate (74% vs. 14%) [23]. Further combinations with other drugs, including BCNU, cyclophosphamide, mitoxantrone, topotecan, and bortezomib, have been explored within small patient cohorts in several phase I/II studies, as well as retrospective studies [24]. Moreover, another phase III study compared tandem high-dose melphalan with total-marrow irradiation, busulfan, and cyclophosphamide (TMI/Bu/Cy). While TMI/Bu/Cy was associated with higher rates of pulmonary and gastrointestinal toxicity, high-dose melphalan showed a more favorable toxicity profile and led to higher CR rates (32.2% vs. 17.5%, *p* = 0.022). However, no differences were found as to event-free survival or OS [26].

A phase II study assessed the safety and efficacy of tandem transplantation with sequential treosulfan and melphalan in MM patients. Grade 3/4 infections and mucositis occurred in 5% of the patients receiving treosulfan [27]. Moreover, a large retrospective study compared conditioning with treosulfan to other regimens prior to allogeneic stem cell transplantation in MM patients. The patients conditioned with treosulfan showed improved PFS and OS [19]. To the best of our knowledge, no previous data are available for TreoMel HDCT in MM. In previous work, we showed comparable toxicity and efficacy profiles for TreoMel vs. BuMel in AML patients undergoing ASCT [20]. The efficacy of the TreoMel HDCT consolidation has also been demonstrated in pediatric patients with metastatic Ewing sarcoma [28]. Currently, a prospective ongoing phase 2 study is assessing the safety and efficacy of TreoMel as compared to melphalan single drug in MM (NCT05636787).

Based on our own AML data, TreoMel is now being used as the standard of care HDCT for fit MM patients at our institution. In the current work, we retrospectively analyzed data from 115 MM patients who received consolidation with TreoMel. In our patient cohort, the median age was 61, and slightly more than half of the patients (63%) were male, which is in line with previous MM studies [9]. Cytogenetic results were available for the majority of patients (91%), with high-risk alterations found in 28% of patients. For the purpose of the efficacy analysis, 1q21 amplification was classified as standard risk [29]. Our patient population was homogeneous regarding treatment history. The majority of patients (83%) received VRd as an induction regimen, and 107 (93%) patients received only one HDCT consolidation.

Regarding the treosulfan pharmacokinetics assessed via mass spectrometry, we observed high interpatient variability, which is in line with our previous AML data [20]. It is not fully clear whether treosulfan distribution shows high interpatient variability at a steady state (V_ss_) due to differences in total body water percentage or age and whether treosulfan AUC correlates with toxicity [20,30,31,32]. Moreover, the treosulfan AUC and peak values were higher in female patients (*p* = 0.001), most probably due to lower glomerular filtration and total body water content [33]. The higher AUC and peak values showed a positive but weak correlation with longer hospitalizations (r = 0.39, *p* = 0.0001). However, prospective studies would be required to assess whether a dose modification could be safely enacted in female patients without compromising drug efficacy. Moreover, remission status post-HDCT and ASCT showed no correlation with treosulfan pharmacokinetics.

Overall, TreoMel showed a favorable safety profile in our MM patient cohort. The median duration of pancytopenia was 8 days, with a highly variable interpatient range between +5 and +24 days. This is comparable to melphalan at 200 mg/m^2^ hematologic toxicity in MM patients [34,35], as well as to TreoMel toxicity in AML patients [20]. Gastrointestinal disorders were frequent, leading to the requirement of parenteral nourishment in 88% of the patients. Moreover, the majority (87%) of patients experienced some grade of malnutrition. Only eight patients (7%) developed reversible acute kidney injury (rAKI). For melphalan at 200 mg/m^2^, the rAKI rates were around 5% for split-dose melphalan and 1% for one-day melphalan [34]. In 2019, we assessed renal toxicity with dose-intensified bendamustine-based HDCT in 122 lymphoma and MM patients, reporting a rather high rAKI rate of 41.8%. This rAKI was mild to moderate in the majority of patients, and only three patients required transient hemodialysis [15]. The infectious complication rate was similar to previously published melphalan data overall [36].

At 31 months, an OS rate of 83% was observed, as well as a median PFS of 30 months. sCR was achieved in 74 (65%) patients, CR in 22 patients (19%), and no patients showed PD as a first response. Within the follow-up period, 23 patients (20%) relapsed and seven (6%) died: four patients due to progression and two due to infectious complications post-HDCT. In our patient cohort, HDCT led to a higher rate of complete responses when compared to previous studies of melphalan at 200 mg/m^2^ [11,12]. Moreover, this rate was comparable to the CR rate reported for the quadruplet combination in the GRIFFIN study [14]. Of relevance, in our study population, only 10% of the patients received quadruplet daratumumab-based induction therapy.

Despite substantial advances in anti-MM treatment, early disease recurrence after induction and consolidation treatment still remains a relevant clinical challenge and most frequently affects patients with high-risk cytogenetic abnormalities, such as del(17p), t(4;14), and t(14;16). In our cohort, 27% of the patients had high-risk cytogenetics. The patients with standard- vs. high-risk cytogenetics had similar response rates. However, a non-significant trend towards poorer PFS and OS was observed for high-risk patients, which is in line with previously published data [37,38].

To the best of our knowledge, this is the first study to assess the efficacy and safety of TreoMel in a large cohort (*n* = 115) of MM patients receiving HDCT consolidation. Despite a retrospective study design, our results constitute a relevant contribution to this novel HDCT regimen in the clinical management of MM patients.

## 5. Conclusions

HDCT consolidation with TreoMel led to an encouraging complete response rate of 84% in a large MM patient cohort, with a median PFS of 30 months and an OS rate of 83% at 31 months follow-up. The safety profile was globally manageable and comparable to 200 mg/m^2^ melphalan data. For patients with high-risk cytogenetics, we observed no significant differences in their response and MRD rates, but a trend towards poorer PFS and OS was observed. Treosulfan pharmacokinetics had no impact on treatment response, but the AUC and peak values were significantly higher in female patients, and higher values correlated with longer hospitalizations. Overall, HDCT with TreoMel showed a promising complete response rate and safety profile in MM patients. Further prospective studies should explore the optimal dosing of treosulfan to optimize efficacy and minimize adverse events.

## Figures and Tables

**Table 1:** Patient baseline characteristics.

Characteristics	All Patients (*n* = 115)
Median age, years (range)	61 (36–73)
Males/females, *n* (ratio)	73/42 (1.74)
Paraprotein subtype, *n* (%)	
kappa light chain	75 (65%)
lambda light chain	40 (35%)
IgG	79 (69%)
IgA	16 (14%)
IgM	1 (1%)
light chain only	19 (16%)
R-ISS Stage *	
I	27 (25%)
II	51 (48%)
III	29 (27%)
MM diagnostic criteria	
Anemia (<110 g/L), *n* (%)	46 (40%)
Hypercalcemia (>2.6 mmol/L), *n* (%)	21 (18%)
Creatinine > 104 µmol/L, *n* (%)	23 (20%)
Osteolytic lesions, *n* (%)	88 (77%)
BM infiltration, median (range)	60% (5–100%)
Cytogenetics	
Available, *n* (%)	91 (79%)
High-risk (t(4;14), t(14;16), del(17p))	25 (28%)
Translocation (4;14)	17 (19%)
Translocation (14;16)	3 (3%)
Deletion (17p)	4 (4%)
Standard-risk	66 (72%)
1q21+	22 (24%)

* Missing data in eight patients.

**Table 2:** Summary of induction, HDCT consolidation, and maintenance therapies.

Parameter	Number (%)
Number of previous treatment lines	
1	107 (93%)
2	5 (4%)
3	3 (3%)
Induction treatment regimens *^a^	
Bortezomib, lenalidomide, dexamethasone	98 (85% *^a^)
Daratumomab, lenalidomide, bortezomib, dexamethasone	11 (10% *^a^)
Bortezomib, cyclophosphamide, dexamethasone	5 (4% *^a^)
Daratumomab, lenalidomide, dexamethasone	3 (3% *^a^)
Daratumomab, carfilzomib, dexamethasone	2 (2% *^a^)
Number of HDCT and ASCT	
1	107 (93%)
2	7 (6%)
3	1 (1%)
Maintenance therapy after HDCT *^b^	
Lenalidomide	97 (92%)
Daratumomab and lenalidomide	2 (2%)
Rituximab and lenalidomide	1 (1%)
None	5 (5%)

*^a^ Some patients received more than one anti-MM regimen; *^b^ no data available for 10 patients.

**Table 3:** Adverse events.

Parameter	Number
Hospitalization days, median (range)	22 (16–85)
Hematologic	
Duration of pancytopenia, median (range)	8 (5–24)
Platelet transfusion required, *n* (%)	109 (95%)
Platelet concentrates transfused, median (range)	3 (1–30)
Erythrocyte transfusion required, *n* (%)	58 (50%)
Erythrocyte concentrates transfused, median (range)	1 (1–19)
Gastrointestinal	
Parenteral nutrition, *n* (%)	101 (88%)
Days of TPN, median (range)	9 (2–25)
Malnutrition (%)	100 (87%)
Neutropenic entercolitis, *n* (%)	72 (63%)
Refeeding syndrome, *n* (%)	44 (38%)
Mucositis, *n* (%)	4 (3%)
Renal	
Acute kidney injury	8 (7%)
Stage * I, *n* (%)	4 (3%)
Stage II, *n* (%)	2 (2%)
Stage III, *n* (%)	2 (2%)
Infections	
Febrile neutropenia, *n* (%)	108 (94%)
Bacteremia, *n* (%)	28 (24%)
Sepsis (SOFA ≥ 3)	4 (3%)
Candidosis, *n* (%)	14 (12%)
Deaths related to infectious complications	2 (2%)
Neurological and mental disorders	
Polyneuropathy, *n* (%)	22 (19%)
Delirium, *n* (%)	5 (4%)
Metabolic disorders	
Steroid induced diabetes, *n* (%)	27 (23%)
Aggravated diabetes type II, *n* (%)	8 (7%)
Hepatic disorders, *n* (%)	7 (6%)
Cardiac disorders	
Atrial fibrillation, *n* (%)	16 (14%)
Transfer to ICU, *n* (%)	4 (3%)

* KDIGO 2012; ICU: intensive care unit; SOFA: sepsis-related organ failure assessment score; TPN: total parenteral nutrition.

**Table 4:** Outcome of treatment consolidation with TreoMel HDCT and ASCT.

Parameter	Number (%)
Best response after HDCT and ASCT *^a^	
sCR	74 (65%)
CR	22 (19%)
VGPR	10 (9%)
PR	8 (7%)
SD	0 (0%)
PD	0 (0%)
Remission status before HDCT and ASCT	
sCR	0 (0%) *^b^
CR	16 (14%)
VGPR	50 (44%)
PR	29 (25%)
SD	5 (4%)
PD	0 (0%)
not available	15 (13%)
Relapse after HDCT and ASCT	23 (20%)
Median PFS, months (range)	30 (0.4–31)
OS, %	83%
Deaths	7 (6%)
due to disease progression	4 (3%)
due to infectious complications	3 (3%)
Median follow-up from start of TreoMel, months (range)	12 (0.4–31)

ASCT: autologous stem cell transplantation; CR: complete response; HDCT: high-dose chemotherapy; OS: overall survival; PD: progressive disease; PFS: progression-free survival; PR: partial response; sCR: stringent complete response; SD: stable disease; VGPR: very good partial remission; *^a^: response data were missing for one patient; *^b^: bone marrow biopsies not systematically performed before HDCT and ASCT.

## Data Availability

The data presented in this study are available upon request from the corresponding author.
